# Comparison of 3D and 2D late gadolinium enhancement magnetic resonance imaging in patients with acute and chronic myocarditis

**DOI:** 10.1007/s10554-020-01966-7

**Published:** 2020-08-13

**Authors:** M. Polacin, I. Kapos, M. Gastl, C. Blüthgen, M. Karolyi, J. von Spiczak, M. Eberhard, B. Baessler, H. Alkadhi, S. Kozerke, R. Manka

**Affiliations:** 1 Institute of Diagnostic and Interventional Radiology, University Hospital Zurich, Raemistrasse 100, 8091 Zurich, Switzerland; 2 Department of Cardiology, University Heart Center, University Hospital Zurich, Raemistrasse 100, 8091 Zurich, Switzerland; 3 Institute for Biomedical Engineering, University and ETH Zurich, Gloriastrasse 35, 8092 Zurich, Switzerland

**Keywords:** Cardiac imaging, Magnetic resonance imaging, Late gadolinium enhancement, Myocarditis

## Abstract

We compared a fast, single breath-hold three dimensional LGE sequence (3D LGE) with an established two dimensional multi breath-hold sequence (2D LGE) and evaluated image quality and the amount of myocardial fibrosis in patients with acute and chronic myocarditis. 3D LGE and 2D LGE (both spatial resolution 1.5 × 1.5 mm^2^, slice-thickness 8 mm, field of view 350 × 350 mm^2^) were acquired in 25 patients with acute myocarditis (mean age 40 ± 18 years, 7 female) and 27 patients with chronic myocarditis (mean age 44 ± 22 years, 9 female) on a 1.5 T MR system. Image quality was evaluated by two independent, blinded readers using a 5-point Likert scale. Total myocardial mass, fibrotic mass and total fibrotic tissue percentage were quantified for both sequences in both groups. There was no significant difference in image quality between 3D und 2D acquisitions in patients with acute (p = 0.8) and chronic (p = 0.5) myocarditis. No significant differences between 3D and 2D acquisitions could be shown for myocardial mass (acute p = 0.2; chronic p = 0.3), fibrous tissue mass (acute p = 0.7; chronic p = 0.1) and total fibrous percentage (acute p = 0.4 and chronic p = 0.2). Inter-observer agreement was substantial to almost perfect. Acquisition time was significantly shorter for 3D LGE (24 ± 5 s) as compared to 2D LGE (350 ± 58 s, p < 0.001). In patients with acute and chronic myocarditis 3D LGE imaging shows equal diagnostic quality compared to standard 2D LGE imaging but with significantly reduced acquisition time.

## Introduction

Cardiac magnetic resonance imaging (MRI) has an exclusive role in the noninvasive detection of myocarditis [1]. Late gadolinium enhancement (LGE) imaging comprises a high sensitivity for the detection of focal fibrotic tissue, which can be commonly found in myocarditis [2]. In acute myocarditis, clinical presentation and outcome strongly vary, ranging from asymptomatic to fulminant myocarditis with acute heart failure and even sudden cardiac death [3–5]. It has been shown that the presence of myocardial fibrosis has diagnostic and prognostic value in patients with acute and chronic myocarditis [6–9]. After acute inflammation has declined, residual myocardial scars can persist and trigger arrhythmias or cardiovascular complications [10]. Therefore diagnosing fibrous tissue is of critical importance, even if symptoms of acute myocarditis have already vanished or have never occurred in the first place.

Cardiac MRI and especially LGE imaging became a key evaluation tool in the challenging diagnosis of myocarditis and prognosis estimation. While current guidelines still propose histological proof as gold standard for diagnosing acute myocarditis, endomyocardial biopsy is rarely performed in everyday clinical practice [11, 12].

Since CMR is used for risk stratification in patients with suspected myocarditis, patients with suspected acute myocarditis should be examined as timely as possible [13–15].

However, due to the relatively long CMR acquisition times and often limited availability of examination slots in the acute hospital setting, further efforts are required to shorten and streamline LGE protocols.

The use of three dimensional (3D), single breath-hold acquisition techniques in LGE imaging has been established in patients with ischemic scars and cardiomyopathies [16–19], offering abbreviated and simplified exams when compared to two dimensional (2D) LGE protocols. However, the value and utility of 3D LGE remains to be demonstrated in patients with myocarditis.

The purpose of this study was the qualitative and quantitative assessment of LGE in patients with acute and chronic myocarditis using a single breath-hold 3D LGE versus standard multiple breath-hold 2D LGE.

## Methods

### Study population

Between July 2017 and September 2018 58 patients (mean age 42 ± 18 years, 16 female) with subepicardial or focal midmyocardial fibrosis were included in this retrospective study. Six male patients that additionally showed subendocardial or transmural scars suspective of coronary artery disease were excluded. Twenty-five of the remaining 52 patients showed focal T2 hyperintensity accompanying scar tissue and presented symptoms of acute myocarditis (chest pain, elevated biomarkers, ECG alterations). The remaining 27 patients were categorized as cases with “chronic myocarditis”, because they displayed scars in subepicardial or focal midmyocardial location without edema (“chronic fibrosis”) and showed no acute clinical symptoms. This study was conducted in accordance to the Declaration of Helsinki and its later amendments and was approved by the institutional review board. All included patients gave written informed consent.

### CMR data acquisition

CMR was performed on a 1.5 T MR system (Achieva, Philips Healthcare, Best, the Netherlands) using a dedicated 5-channel phased array coil. All data were acquired during breath holding in end expiration. After scout and reference scans, functional and geometric assessment was performed using cine balanced steady- state free precession (SSFP) images in standard long- axis geometries (two-, three- and four-chamber view) as well as in short-axis orientation covering the entire left ventricle (LV) (field of view: 350 × 350 mm^2^, matrix: 300 × 300, repetition time/echo time: 3.0/1.5 ms, in-plane resolution, 1.5 × 1.5 mm^2^; number of cardiac phases: 20, section thickness: 8 mm). Edema- sensitive black blood T2-weighted images with and without fat saturation in five short- axis slices were acquired for visualizing myocardial edema [20].

2D LGE images covering the entire LV were acquired 15 min after administration of a bolus of 0.2 mmol of gadobutrol (Gadovist; Bayer Schering Pharma, Zurich, Switzerland) per kilogram body weight. 2D LGE images were acquired in short-axis views by using an inversion recovery gradient-echo sequence: field of view: 350 × 350 mm^2^; matrix: 256 × 256; repetition time/echo time: 7.4/4.4; inversion time: 190–270 ms (individually optimized with a Look-Locker sequence); flip angle: 20°; in-plane resolution: 1.5 × 1.5 mm^2^; section thickness: 8 mm. 3D LGE images were acquired with the following parameters: field of view: 350 × 350 mm^2^; matrix: 256 × 256; repetition time/echo time: 3.6/1.8; inversion time: 190–270 ms (individually optimized with a Look- Locker sequence); flip angle: 15°; in-plane resolution: 1.5 × 1.5 mm^2^; section thickness: 8 mm.

2D and 3D LGE sequences were performed in random order and the acquisition times for both sequences were measured and noted.

### CMR data analysis

For all quantitative analyses, commercially available software (IntelliSpace Portal, Philips, Version 8.0.3) was used. CMR data analysis assessment was performed by an experienced radiologist (4 years of experience in cardiac imaging), blinded to patient characteristics (Table 1). For the acquisition of cardiac volume and function, endocardial contours were drawn in end-systolic and end-diastolic short-axis balanced SSFP images excluding papillary muscle. Subjective image quality analysis and quantitative cardiac MR imaging data analysis of 2D and 3D LGE images were performed in random order. For 33 randomly chosen patients, quantitative analysis of 2D and 3D LGE images was repeated by the first reader after 4 months to assess intra-observer agreement and by a second reader (3 years of experience in cardiac imaging) to assess inter-observer agreement.

**Table 1:** Demographic characteristics

	Acute (n = 25)	Chronic (n = 27)	p-values
Patient demographics			
Sex (male/female)	15/5	17/4	0.6
Age (years)	40 ± 19 [21–72]	43 ± 17 [17–74]	0.7
Height (m)	1.77 ± 0.07 [1.68–1.94]	1.75 ± 0.1 [1.6–1.9]	0.3
Weight (kg)	77.8 ± 12 [59–102]	80.3 ± 17 [56–110]	0.7
BMI	25 ± 3 [8–30]	26 ± 4 [20–36]	0.3
Left ventricular morphology			
LVEDV (ml, 117–200)	168 ± 47 [114–288]	156 ± 37 [81–215]	0.6
LVESV (ml, 31–76)	81 ± 41 [38–195]	67 ± 24 [31–117]	0.4
LVSV (ml, 77–133)	87 ± 18 [57–115]	90 ± 18 [55–115]	0.5
LVEF (%, > 54)	54 ± 11 [26–69]	58 ± 8 [39–68]]	0.3
LV mass (g, 51–87)	57 ± 17 [35–111]	52 ± 12 [34–72]	0.5

*BMI* body mass index, *LVEDV* left ventricular end-diastolic volume, *LVESV* left ventricular end-systolic volume, *LVSV* left ventricular stroke volume, *LVEF* left ventricular ejection fraction; values in round brackets are standard, cohort specific LV values; values in square brackets represent the value range

### Image quality scoring

The image quality of each dataset was graded by the same two blinded independent readers using a five-point Likert scale, in which a score of 1 indicated excellent, a score of 2 good, a score of 3 moderate, a score of 4 poor and a score of 5 nondiagnostic image quality. Reference standard was the 2D LGE image. In case of a Likert score greater than 1 (score: 2–5) the reason for impaired image quality was noted and categorized as being due to motion artifacts, inadequate myocardial nulling, low contrast/high noise or folding artifacts.

### Quantitative evaluation

A semi-automatic approach was used to quantify the amount of LGE. Endocardial and epicardial contours were manually drawn on 2D and 3D LGE images and ROIs were placed in hyperenhanced and normal appearing remote myocardium by the software and reviewed by the reader. Subsequently, the areas of unenhanced and hyperenhanced myocardium were automatically segmented by using a full-width at half-maximum algorithm [21]. After segmentation, myocardial and fibrous tissue masses were calculated and the percentage of fibrous tissue mass relative to total myocardial LV mass was computed. The calculated areas of fibrosis as well as myocardial tissue mass were double-checked by the reader and focal corrections could be performed manually if necessary.

### Statistical analyses

Continuous data are expressed as means ± standard deviations, and categoric data are expressed as numbers and percentages. Image quality scores of the 2D and 3D data sets were compared by using the Wilcoxon signed rank test. Two-tailed paired *t*-tests were used to compare normally distributed continuous data and the Wilcoxon signed rank was used for non-parametric distributed continuous data where appropriate. Agreement between the results of the two acquisition techniques regarding total myocardial mass, fibrous mass and percentage of fibrous mass was assessed by using the Pearson correlation coefficient and Bland–Altman analysis with calculation of the limits of agreement (± 1.96 standard deviation). Intra- and inter-observer variability were assessed by calculating Cohen’s kappa coefficients. Kappa coefficients were considered as follows: ‘slight’ < 0.21, ‘fair’ 0.21–0.4, ‘moderate’ 0.41–0.60, ‘substantial’ 0.61–0.80 and ‘almost perfect’ 0.81–1.0 [22]. All statistical analyses were performed using commercially available software (IBM SPSS Statistics, release 25.0; SPSS, Armonk, NY). Statistical significance of difference was assumed for a p-value below 0.05.

## Results

### Qualitative image analysis

**Fig. 1:**
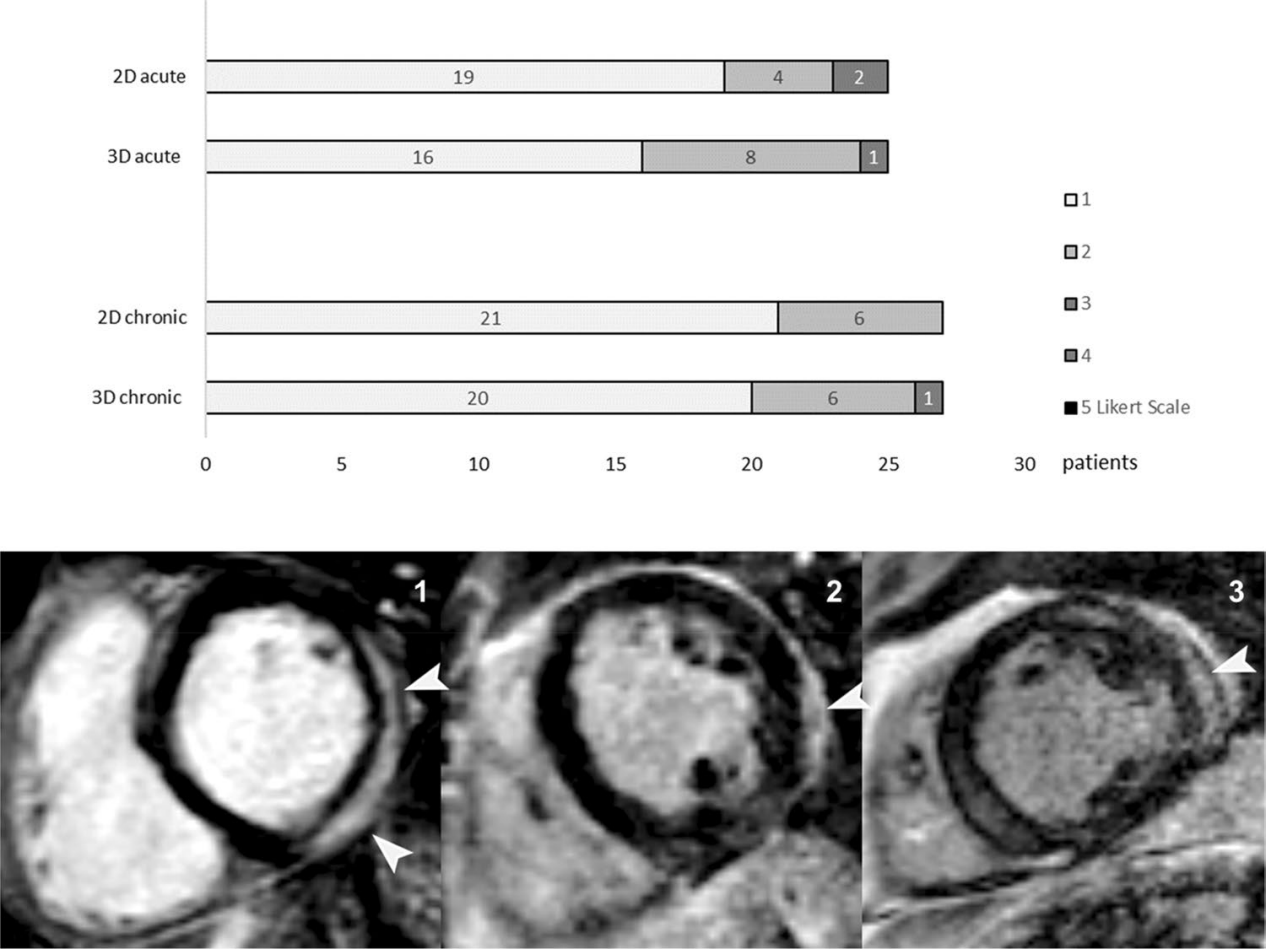
Upper part: Image quality of 3D and 2D LGE datasets of patients with acute myocarditis and chronic inflammatory scars assessed on a 5- point Likert scale from 1 (= excellent image quality) to 5 (= non-diagnostic). Lower part: Short-axis 3D LGE images of three different patients with epicardial fibrosis (arrowheads). Left image demonstrates excellent image quality (= 1), middle image good image quality (= 2, subtle motion artifacts) and right image moderate image quality (= 3, inadequate myocardial nulling, fibrosis still detectable). Poor (score: 4) or non-diagnostic (score: 5) image quality was found in neither acute nor chronic datasets

**Fig. 2:**
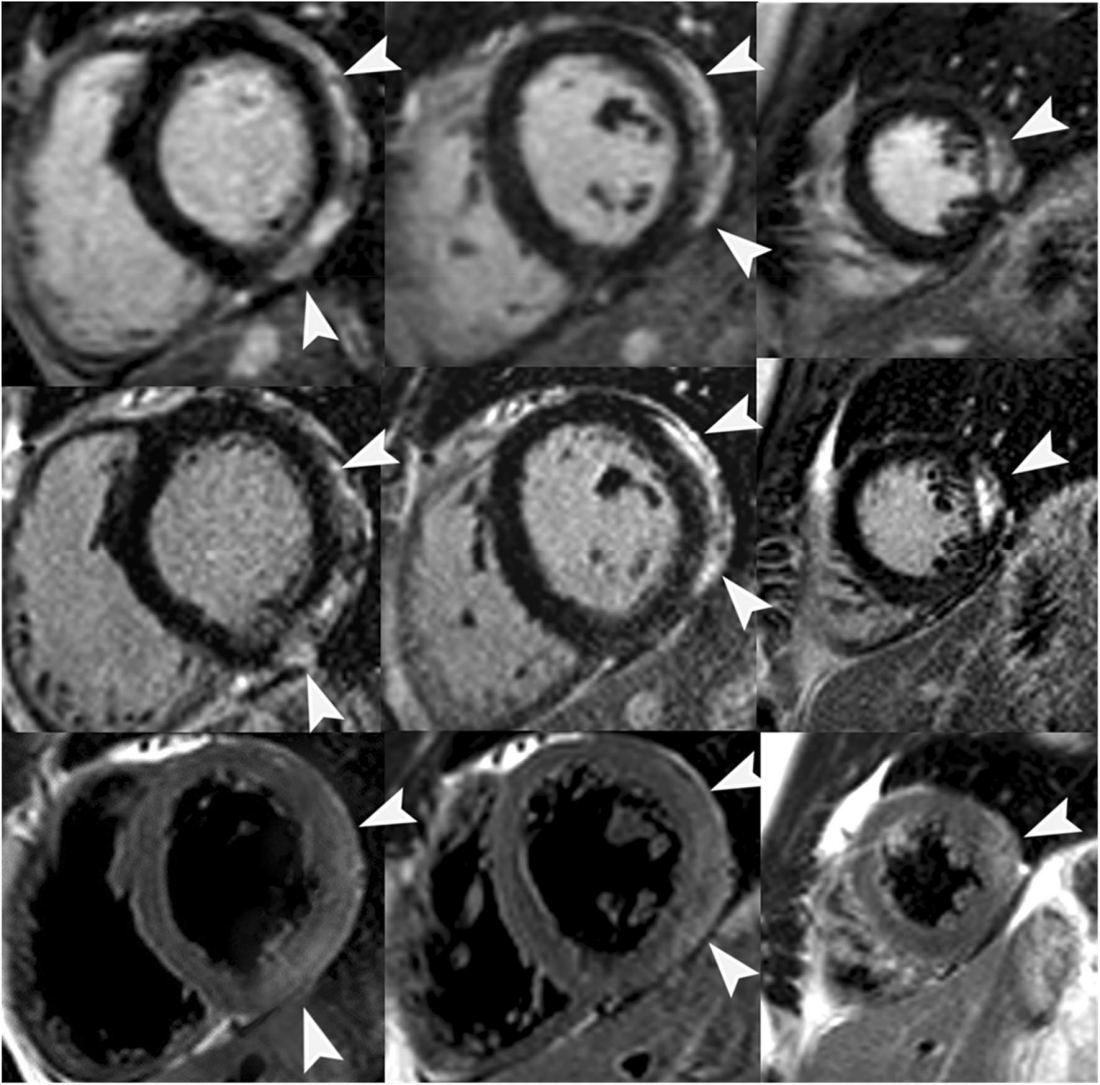
Short-axis LGE CMR images of a 23-year-old male patient with acute myocarditis**.** The upper panel shows basal, midventricular and apical sections from 3D LGE acquisition, the middle panel shows the same slices from 2D LGE acquisition and the lower panel shows the same slices in a T2 weighted sequence. High signal intensity in LGE images (arrowheads) indicating epicardial fibrous tissue in basal and midventricular anterolateral/inferolateral segments as well as apical lateral segment could be noted in both acquisitions with concomitant hyperintense signal in T2-weighted images

In patients with acute myocarditis, 16 out of 25 (64%) from the 3D datasets and 19 out of 25 (76%) from the 2D datasets had excellent image quality (Likert score: 1) (Figs. 1, 2). Average image quality did not vary significantly between 3D and 2D acquisitions (1.4 ± 0.6 vs. 1.3 ± 0.5, p = 0.8). Inter-observer agreement was substantial (κ = 0.76 and 0.71 for 3D and 2D, respectively).

**Fig. 3:**
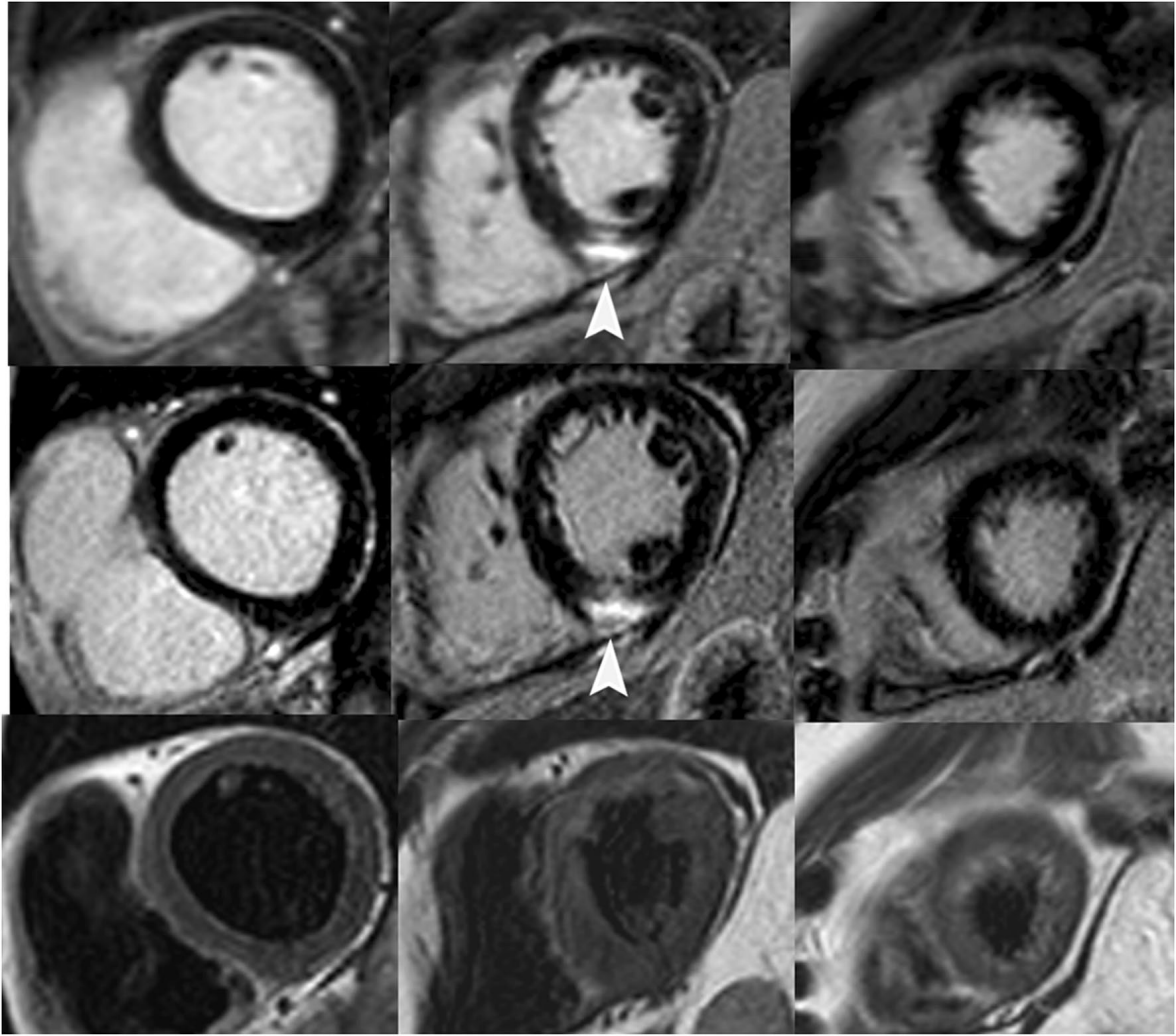
Short-axis LGE CMR images of a 47-year-old male patient with chronic myocarditis**.** The upper panel shows basal, midventricular and apical sections from 3D LGE acquisition, the middle panel shows the same slices from 2D LGE acquisition and lower panel shows the same slices in a T2 weighted sequence. High signal intensity in LGE images (arrowheads) indicating epicardial fibrous tissue in basal and midventricular inferior segments could be noted in both acquisitions with no signal alterations in T2-weighted images

In patients with chronic fibrosis, 20 out of 27 (74%) from the 3D datasets and 21 out of 27 (78%) from the 2D datasets had excellent image quality (Likert score: 1) (Figs. 1 and 3). There was no significant difference in average image quality between 3D and 2D acquisitions (1.3 ± 0.7 vs. 1.2 ± 0.5, p = 0.5). Inter-observer agreement was substantial to almost perfect (κ = 0.76 and 0.81 for 3D and 2D, respectively).

Poor (score: 4) or non-diagnostic (score: 5) image quality was found in neither acute nor chronic datasets. Impaired image quality (Likert score 2 and 3) occurred most frequently due to inadequate myocardial nulling and was as frequent in 3D as in 2D datasets (12/52 and 11/52, respectively).

### Quantitative image analysis

#### Acute myocarditis

There were no significant differences between 3D and 2D acquisitions for myocardial mass (111.9 g ± 33.7 vs. 111.4 g ± 34.1, p = 0.2), fibrous tissue mass (9.2 g ± 5.3 vs. 9.2 g ± 5.2, p = 0.7) and total fibrous percentage (8.3% ± 4.8 vs. 8.2% ± 4.6, p = 0.4) (Table 2) with substantial inter-observer agreement (κ = 0.84, 0.76 and 0.74, respectively) and intra-observer agreement (κ = 0.82, 0.80 and 0.79, respectively).

**Table 2:** Quantitative measurements

	3D	2D	p-values
Acute			
Myocardial mass (g)	111.9 ± 33.7	111.4 ± 34.1	0.2
Fibrous tissue mass (g)	9.2 ± 5.3	9.2 ± 5.2	0.7
Total fibrous percentage (%)	8.3 ± 4.8	8.2 ± 4.6	0.4
Chronic			
Myocardial mass (g)	108.7 ± 25.9	109.1 ± 25.8	0.3
Fibrous tissue mass (g)	5.1 ± 4.7	5.1 ± 4.6	0.1
Total fibrous percentage (%)	4.4 ± 4.3	4.3 ± 4.1	0.2
Acquisition time (s)	24 ± 5	350 ± 58	< 0.001

**Fig. 4:**
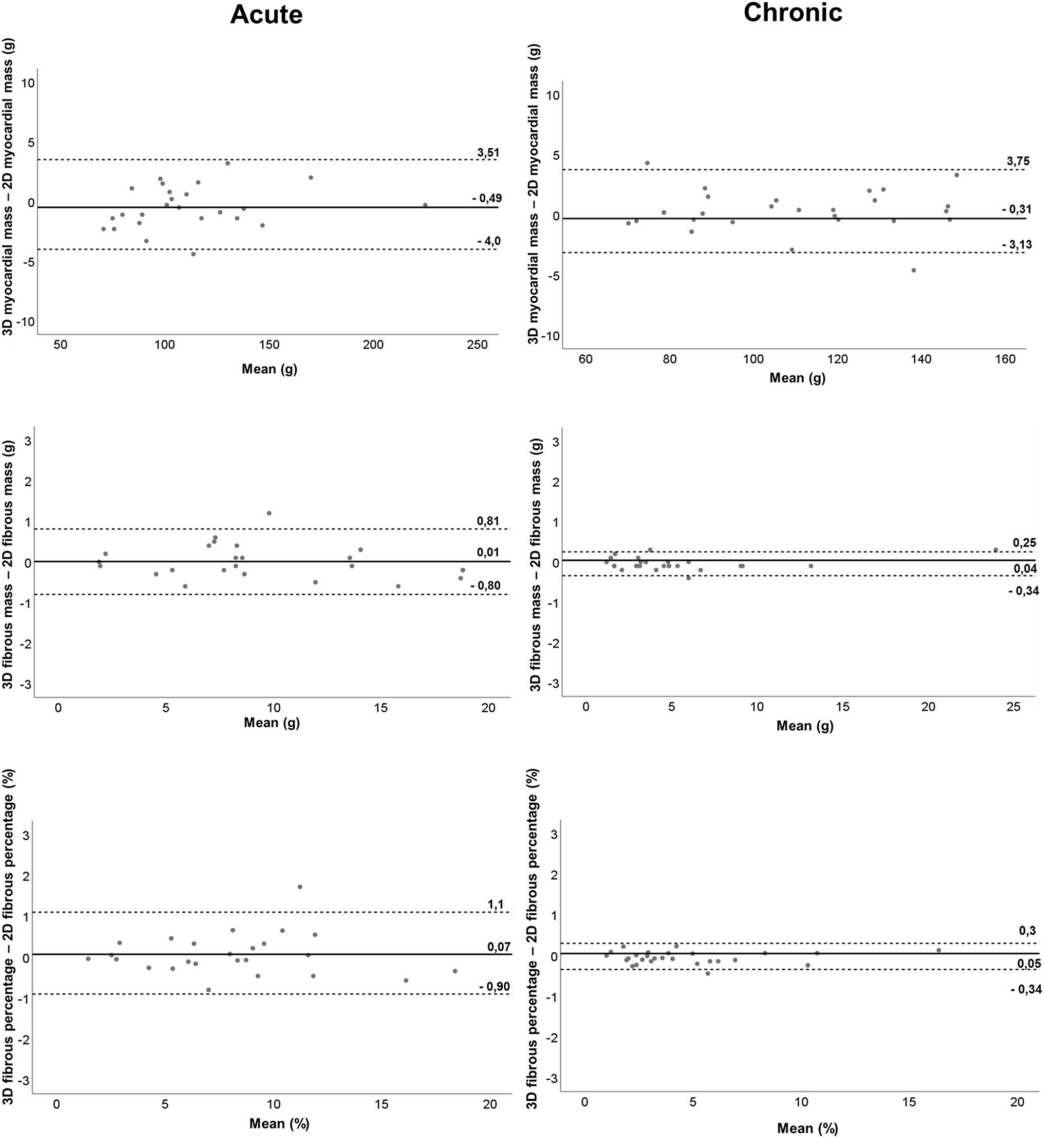
Bland–Altmann plots depicting the mean difference between 3D and 2D acquisitions and corresponding limits of agreement (± 1.96 standard deviation; dotted lines) for myocardial mass, fibrous mass, and fibrous percentage in patients with acute (right) and chronic myocarditis (left)

A significant correlation was found between 3D and 2D datasets for myocardial mass (r = 0.91, p = 0.001), fibrous tissue mass (r = 0.99, p = 0.001) and total fibrous percentage (r = 0.95, p = 0.001). Bland–Altman analysis showed good agreement between 3D and 2D datasets for myocardial mass (mean difference: − 0.49 g; limits of agreement: − 4.0 to 3.51 g), fibrous tissue mass (mean difference: 0.01 g; limits of agreement: − 0.8 to 0.81 g) and total fibrous percentage (%) (mean difference: 0.07%; limits of agreement: − 0.9 to 1.1%) (Fig. 4*).*

#### Chronic myocarditis

There were no significant differences between 3D and 2D acquisitions for myocardial mass (108.7 g ± 25.9 vs. 109.1 g ± 25.8, p = 0.3), fibrous tissue mass (5.1 g ± 4.7 vs. 5.1 g ± 4.6, p = 0.1) and total fibrous percentage (4.4% ± 4.3 vs. 4.3% ± 3.1, p = 0.2) (Table 2) with substantial or almost perfect inter-observer agreement (κ = 0.75, 0.81 and 0.70, respectively) and intra-observer agreement (κ = 0.78, 0.83 and 0.81, respectively).

A significant correlation was found between 3D and 2D datasets for myocardial mass (r = 0.97, p < 0.001), fibrous tissue mass (r = 0.96, p < 0.001) and total fibrous percentage (r = 0.96, p = 0.001). Bland–Altman analysis showed good agreement between 3D and 2D datasets for myocardial mass (mean difference: 0.31 g; limits of agreement: − 3.13 to 3.75 g), fibrous tissue mass (mean difference: 0.04 g; limits of agreement: − 0.34 to 0.25 g) and total fibrous percentage (mean difference: 0.05%; limits of agreement: − 0.34 to 0.3%) (Fig. 4).

#### Acquisition time

Acquisition time was significantly shorter for 3D LGE (24 ± 5 s) as compared to 2D LGE (350 ± 58 s, p < 0.001).

## Discussion

This study compared a fast, single breath-hold 3D LGE imaging sequence with a clinically established multi breath-hold 2D LGE sequence in patients with acute and chronic myocarditis. In both groups 3D LGE showed equivalent image quality and quantitative information in comparison with the 2D LGE sequence but with significantly reduced acquisition time. Single breath-hold 3D LGE imaging has been established for ischemic scars and cardiomyopathies, providing comparable image quality to multi breath-hold 2D LGE [18, 23, 24].

However, fibrosis in inflammatory disease has lower signal intensity compared with ischemic scars, with usually smaller amounts of fibrotic tissue in typically subepicardial location and lack of concomitant wall motion abnormalities [25, 26]. Optimal image quality of LGE images is of utmost importance in order not to miss these subtle areas of fibrosis and the established multi breath-hold 2D LGE sequences are still preferably used in myocarditis protocols [27–29].

All fibrotic areas in patients with acute and chronic myocarditis in 2D LGE were visible in 3D LGE. In patients with acute and chronic myocarditis image quality in 3D datasets was comparable to 2D datasets and inter-observer agreement was substantial to almost perfect. Impaired image quality was mostly due to inadequate myocardial nulling and occurred with same frequency in 3D and 2D datasets.

Quantitative LGE evaluation between 3D and 2D datasets in both patient groups showed no significant differences with substantial to almost perfect inter- and intra-observer agreement, even though some patients—especially those with chronic inflammatory scars—had subtle LGE areas with very low scar burden.

For a short-axis one breath-hold 3D LGE sequence acquisition time was approximately 24 s compared to nearly 6 min for a multi breath-hold 2D acquisition, this is a time reduction of more than 90%. Reduced scan time serves patient comfort and is economically favorable, especially in regard to patients that will receive follow-up MRIs. Moreover, if image quality of LGE is low, e.g. due to wrong inversion time, 3D LGE can easily be repeated without significantly prolonging overall exam times.

Some limitations have to be mentioned. 2D LGE was considered the reference standard for qualitative and quantitative LGE evaluation, but histological proof of LGE areas was not conducted. LGE appearance (subepicardial or focal midmyocardial fibrosis) in 2D LGE and T2-weighted image, respectively, was the main criterion for study inclusion and patients with a history of ischemic heart disease or other non-ischemic cardiomyopathies have not been explicitly excluded.

In conclusion, single breath-hold 3D LGE shows equal diagnostic quality in acute and chronic inflammatory scars compared to standard 2D LGE imaging at significantly reduced acquisition time. Therefore, single breath-hold 3D LGE can be used instead of multi breath-hold 2D LGE in patients with acute and chronic myocarditis.

## Abbreviations

2D: Two dimensional
3D: Three dimensional
ECG: Electrocardiogramm
LGE: Late gadolinium enhancement
LV: Left ventricle/left-ventricular
LVEDV: Left ventricular end-diastolic volume
LVEF: Left ventricular ejection fraction
LVESV: Left ventricular end-systolic volume
LVSV: Left ventricular stroke volume
MR/MRI: Magnetic resonance/magnetic resonance imaging
ms: Milliseconds
s: Seconds
SSFP: Steady-state free precession
T: Tesla
